# Surgery of the Peritoneum, etc.

**Published:** 1903-09-12

**Authors:** 


### SURGERY OF THE PERITONEUM, ETC.

**Acute Septic Peritonitis.**—Sheild [1] says there are two especial localities where septic wounds of the peritoneum are peculiarly obscure and concealed from view; one is through the vagina or uterus, the other is by the rectum. The severity of the initial symptoms may often with justice be looked upon as a most important diagnostic point in estimating the gravity and extent of the perforating lesion and the peritonitis likely to ensue. The vast majority of cases of acute septic peritonitis originate in infection from the alimentary canal, and in children and young adults a sudden acute and dangerous peritonitis is far more likely to be due to perforation of the appendix than anything else. Next in frequency come the perforations of the stomach and duodenum. These dangerous lesions form a significant proportion of causes of acute peritonitis in adult life. In women are superadded lesions and diseases which occur about the pelvic generative organs. Thus the rupture of an ovarian abscess or suppurating cyst, the sudden extension of infection from a suppurating tube, are familiar examples of causes of peritonitis of pelvic origin, often difficult and dubious enough in actual practice to diagnose and verify. Among more exceptional causes may be mentioned the perforation of typhoid fever, suppurations and perforations of the gall-bladder, the rupture of hydatid cysts, the extravasation of hepatic, spinal, or glandular abscesses into the abdominal cavity, and leakage in connection with cancer of the large intestine. Any period of delay beyond six hours in perforated septic peritonitis means the possible destruction of the patient. The following rules of practice may be formulated:—1. Acute peritonitis is the result of infection, and the lesion which produces it usually needs surgical treatment. 2. The spreading of peritonitis over a larger and larger area, no limiting mass of inflammatory nature being detected, is a serious symptom, and calls for operation. 3. The treatment of acute peritonitis without operation is generally of the "hap-hazard" type, though recovery may take place if the infection be slight. 4. For purposes of a practical diagnosis the abdomen may be divided into two zones—an upper and a lower. 5. In the upper zone the cause of the acute peritonitis may be in the stomach, duodenum, or gall-bladder; in the lower zone the appendix, or the pelvic generative organs in women. 6. Anomalous and rare causes of peritonitis, as the rupture of a spinal abscess, or a hydatid cyst, will seldom be correctly diagnosed without exploratory operation.

**Drainage after Abdominal Section.**—McGuire[2] calls attention to the following facts in connection with this subject:—1. The peritoneum can absorb large quantities of fluid, sometimes a weight equivalent to that of the animal in 24 hours. 2. Irritation or inflammation of the peritoneum lessens its absorptive power. 3. The peritoneum can neutralise large numbers of pathogenic germs without the development of peritonitis. 4. The more rapid the absorption from the peritoneum the greater the toleration to bacteria. 5. Stagnation of fluid in the peritoneal cavity favours the development of peritonitis. 6. Leucocytes carry foreign particles from the peritoneal cavity to the lymph and blood vessels. 7. There is a current in the peritoneum which carries fluid and foreign particles towards the diaphragm. Thus it will be seen that the peritoneal cavity, up to a certain point, drains itself. After that point is passed it ceases to drain at all. Despite the labour devoted to modern surgical technique, absolute asepsis is an unattained ideal, and an abdomen opened is an abdomen infected. All require drainage, and the question is simply to determine the dividing line between the cases that may safely be left to nature and those which require artificial aid. The objections to the employment of a drain are that it is a foreign body; prevents primary union; endangers secondary infection; sometimes causes fæcal fistula; frequently gives pain, and always prolongs convalescence. Broadly stated, drainage should be used in the following cases:—1. When bleeding is uncontrollable, as after the enucleation of an intra-ligamentary cyst. 2. When profuse serous transudation is probable, as after the removal of a large adherent ovarian tumour. 3. When localised collections of pus are found, as in a case of appendicular abscess. 4. When acute general peritonitis exists, as in a case of perforating ulcer of typhoid. 5. When protective adhesions are desired, as in drainage of the gall-bladder. 6. When secondary infection is feared, as in some cases of intestinal lesions. Drainage of the abdominal cavity may be effected in three ways; by natural drainage, by indirect drainage, and by direct drainage. Natural drainage is dependent on the absorptive power of the peritoneum and the phagocytic action of the leucocytes, and should be maintained by injuring the tissues as little as possible. Indirect drainage is merely an artificial increase of natural drainage, and it may be effected either by saline purgation, or by postural drainage—the foot of the bed being elevated some 18 inches so that gravity may carry any free fluid to the under side of the diaphragm, where its absorption is more rapid than from other portions of the peritoneum. Direct drainage is the abstraction of fluid from the cavity by tubes, or gauze, or wicks, which are introduced either through the primary wound or through secondary incision. Drainage by means of gauze strips has largely supplanted the use of the tube. Gauze not only effects drainage by capillary action, but may also be employed to arrest hæmorrhage by pressure, to isolate an infected region from the general peritoneal cavity by acting as a wall or barrier, and to promote the formation of adhesions in cases where they are essential to safety. Mikulicz's drain consists of a square of gauze with a string fastened to its centre. If it is taken by the four corners it forms a pouch or bag with the string coming through its mouth. This bag is introduced into the abdominal cavity, and then packed with strips of gauze. The author's drain consists of a soft gum tube surrounded by numerous strands of cordine, both enclosed in a thin rubber protective.

**The Prevention of Ventral Hernia after Laparotomy** is, according to Hahn,[3] best effected by careful suturing of the external wound. Special importance is attached to the following details of his method:—(1) The peritoneal layer, together with the fascia transversalis, should be sutured quite apart from the other layers of the abdominal wall; (2) so far as possible aponeurotic and not muscular parts of the different layers should be stitched, the latter when brought together by light sutures are, it is stated, invariably torn through; (3) in a corpulent subject the thick layer of subcutaneous fat should be sutured by very fine silk. The author prefers perfectly sterilised silk as the material for sutures; it is cheap, can be readily manipulated, is non-absorbable, and remains, as it were, a portion of the living tissue.

**Peritoneal Adhesions**, by fixing various parts of the colon, may lead to imperfect emptying of its contents and simulation of the symptoms of appendicitis or movable kidney. Lane[4] says that the symptoms presented by a right movable kidney and by an obstructed hepatic flexure are more alike than is generally supposed. They may both give a feeling of dragging in the loin, tenderness on firm pressure below the last rib posteriorly, a feeling of fulness or tenderness or pain in the right iliac fossa, especially towards the end of the day or after the erect posture has been assumed for some considerable time. In both cases there are occasional exacerbations of these pains, which may spread to the whole abdomen and may be associated with a feeling of nausea or even of vomiting. Both conditions may be relieved by purgation. By the assumption of the supine position relief is often obtained in both—in the one case by the relief of the urinary and venous engorgement of the kidney, and in the other by the strain being taken off the newly-developed colic mesentery and adhesions, by means of which the lumen of the hepatic flexure is freed and the exit of the contents of the cesspool facilitated. A feature of some assistance in the case of a painful movable kidney is a tenderness of its ureter, which can be best detected by pressure on this structure as it crosses the psoas and the iliac vessels.

**A Removable Deep Suture.**—Milton[5] suggests a form of suture modelled after the "lock-stitch" of a sewing machine. The method of introducing the suture is of the simplest. All that is required is a mounted needle with an eye near the point; through this is threaded a long piece of silk. This forms the primary thread; the secondary thread is best formed by a stout piece of silkworm gut which is sufficiently rigid to avoid kinking and being drawn into the sutured tissue. Supposing the peritoneum is to be sutured after a laparotomy the procedure will be as follows:—The two layers of the peritoneum which are to be sewn together are defined; the mounted needle, threaded with the long end of the thread on the left hand or upper side of the needle when held as it will be when piercing the tissue, is passed from the operator through both layers of the peritoneum, beginning at the lower angle of the wound. The needle is then withdrawn, leaving a loop of silk protruding through the layers of peritoneum on the side of the assistant; through this loop the assistant passes the end of the silkworm gut, from below upwards, and the loop is withdrawn flush with the assistant's side of the united flaps. The needle, not having been unthreaded, is again thrust through the flaps for the second stitch and again withdrawn, leaving a loop as in the first instance; the silkworm gut is threaded through this second loop and the process is repeated till the whole of the flaps are united. The needle is then unthreaded, leaving a fairly long end to the silk. The four ends, two silk and two silkworm gut, are each in turn threaded on an ordinary needle and made to pierce the skin on its own side of the wound, and are left thus whilst the wound in the abdominal wall is sutured in the ordinary way. This having been done the two lower ends of the peritoneal suture, being one of silk and the other of silkworm gut, are then tied together and cut to a convenient length. The same is done with the two ends at the upper end of the wound, and the process is complete. When the wound is healed the stitch can be removed by cutting the knots, pulling out first the silkworm-gut stitch which will offer no resistance, and then the silk stitch, which having lost its support, can be pulled out with equal facility.

[1] Clin. Jour., May 20, 1903, p. 65. [2] Charlotte Med. Jour., Dec., 1902, p. 420. [3] Brit. Med. Jour., Feb. 28, 1903, Epitome No. 145. [4] Clin. Jour., March 25, 1903, p. 354. [5] Lancet, June 6, 1903, p. 1585.

